# Does coaching matter? Examining the impact of specific practice facilitation strategies on implementation of quality improvement interventions in the Healthy Hearts in the Heartland study

**DOI:** 10.1186/s13012-021-01100-8

**Published:** 2021-03-31

**Authors:** Theresa L. Walunas, Jiancheng Ye, Jennifer Bannon, Ann Wang, Abel N. Kho, Justin D. Smith, Nicholas Soulakis

**Affiliations:** 1 Department of Medicine, Division of General Internal Medicine and Geriatrics, Northwestern University Feinberg School of Medicine, Chicago, IL USA; 2 Center for Health Information Partnerships, Institute for Public Health and Medicine, Northwestern University Feinberg School of Medicine, 625 N. Michigan, 15th Floor, Chicago, IL 60611 USA; 3 Department of Preventive Medicine, Division of Healthcare and Biomedical Informatics, Northwestern University Feinberg School of Medicine, Chicago, IL USA; 4 Department of Population Health Science, University of Utah School of Medicine, Salt Lake City, UT USA

**Keywords:** Practice facilitation, Quality improvement, Cardiovascular care, Primary care, Strategy framework

## Abstract

**Background:**

Practice facilitation is a multicomponent implementation strategy used to improve the capacity for practices to address care quality and implementation gaps. We sought to assess whether practice facilitators use of coaching strategies aimed at improving self-sufficiency were associated with improved implementation of quality improvement (QI) interventions in the Healthy Hearts in the Heartland Study.

**Methods:**

We mapped 27 practice facilitation activities to a framework that classifies practice facilitation strategies by the degree to which the practice develops its own process expertise (Doing Tasks, Project Management, Consulting, Teaching, and Coaching) and then used regression tree analysis to group practices by facilitation strategies experienced. Kruskal-Wallis tests were used to assess whether practice groups identified by regression tree analysis were associated with successful implementation of QI interventions and practice and study context variables.

**Results:**

There was no association between number of strategies performed by practice facilitators and number of QI interventions implemented. Regression tree analysis identified 4 distinct practice groups based on the number of Project Management and Coaching strategies performed. The median number of interventions increased across the groups. Practices receiving > 4 project management and > 6 coaching activities implemented a median of 17 of 35 interventions. Groups did not differ significantly by practice size, association with a healthcare network, or practice type. Statistically significant differences in practice location, number and duration of facilitator visits, and early study termination emerged among the groups, compared to the overall practice population.

**Conclusions:**

Practices that engage in more coaching-based strategies with practice facilitators are more likely to implement more QI interventions, and practice receptivity to these strategies was not dependent on basic practice demographics.

**Supplementary Information:**

The online version contains supplementary material available at 10.1186/s13012-021-01100-8.

Contributions to the literature

- Practice coaching is a key strategy for supporting implementation of quality improvement interventions in small primary care practices.
- The practice facilitation strategy “spectrum” is an effective foundation for translating study-specific activities to a common framework that can be used to assess the relationship of the strategies to study outcomes within and across projects.
- It is possible to develop an informatics-driven framework and terminology system that can be used for cross-study comparison of the impact of practice facilitation strategies on intervention implementation across diverse practice settings.

## Background

To deliver high-quality, efficient, patient-centered care, many healthcare networks and payers expect practices to implement continuous quality improvement (QI) efforts. However, small primary care practices face challenges due to lack of staff and resources to invest in infrastructure and training that are essential elements of QI capacity [1–5]. Practice facilitation is an emerging implementation strategy aimed at assisting practices with developing capacity for sustained implementation of QI interventions [6, 7]. Recent studies demonstrate that practice facilitation increases the likelihood of success of QI initiatives, increases provider adherence to evidence-based guidelines, and improves care quality metrics in a broad array of clinical settings [8–11]. Practice facilitation has been used to effectively support implementation of evidence-based behavioral health interventions [12, 13], integrated behavioral health and primary care [14], colorectal screening [15], antibiotic stewardship [16], pediatric preventive care [11], and implementation of patient-centered medical home programs [17–19].

Even with increasing adoption, there is little evidence of what constitutes effective practice facilitation [8]. Practice facilitators perform a variety of roles and activities tailored to the intervention and practice [6, 8, 20]. Depending on context, they may act as technical assistance, data collectors, project managers, liaisons, consultants, teachers, and coaches [6, 21]. Few conceptual frameworks or standardized terminologies exist to describe the specific implementation strategies constituting practice facilitation. Such a framework is needed to support comparison of practice facilitation within and across initiatives. The lack of such a framework renders it difficult to ascertain which facilitation strategies are broadly useful for sustained implementation of QI and other interventions. Thus, while previous reviews have shown a relationship between practice facilitation intensity and outcomes [8, 9], it remains to be determined which approaches are most conducive to successful practice transformation and what data elements are most essential for evaluation of practice facilitation strategies and activities and development of models to better understand the interplay of practice facilitators and practices in quality improvement success. There is a need to open the “black box” of practice facilitation to better understand the active ingredients of this strategy that are responsible for the observed positive changes. From this line of inquiry, better understanding of the mechanisms of action are possible.

The shortcomings described that pertain to practice facilitation are not unique to this specific implementation strategy. In recent years, there have been numerous calls to better specify, define, and describe implementation strategies [22, 23]. This issue applies to all implementation strategies but is particularly germane to multicomponent or blended strategies (as opposed to discrete strategies), which by definition comprise more than one activity [24]. The precise composition of the strategies used within a blended strategy “package” can and often do vary by implementation site or by the actor who enacts them. Yet, the variability itself and the reasons for it are often poorly understood and often go unreported in sufficient detail to be empirically examined at the component level [25, 26]. Practice facilitation is a prime demonstration case for the development and validation of methods that better capture the nature and effects of a multicomponent strategy. Such methods could then be applied to understand the degree of variation between sites and clusters within projects, and compare them between projects, as well as understand the relationship with adoption and sustained change that results from practice facilitation.

**Fig. 1:**
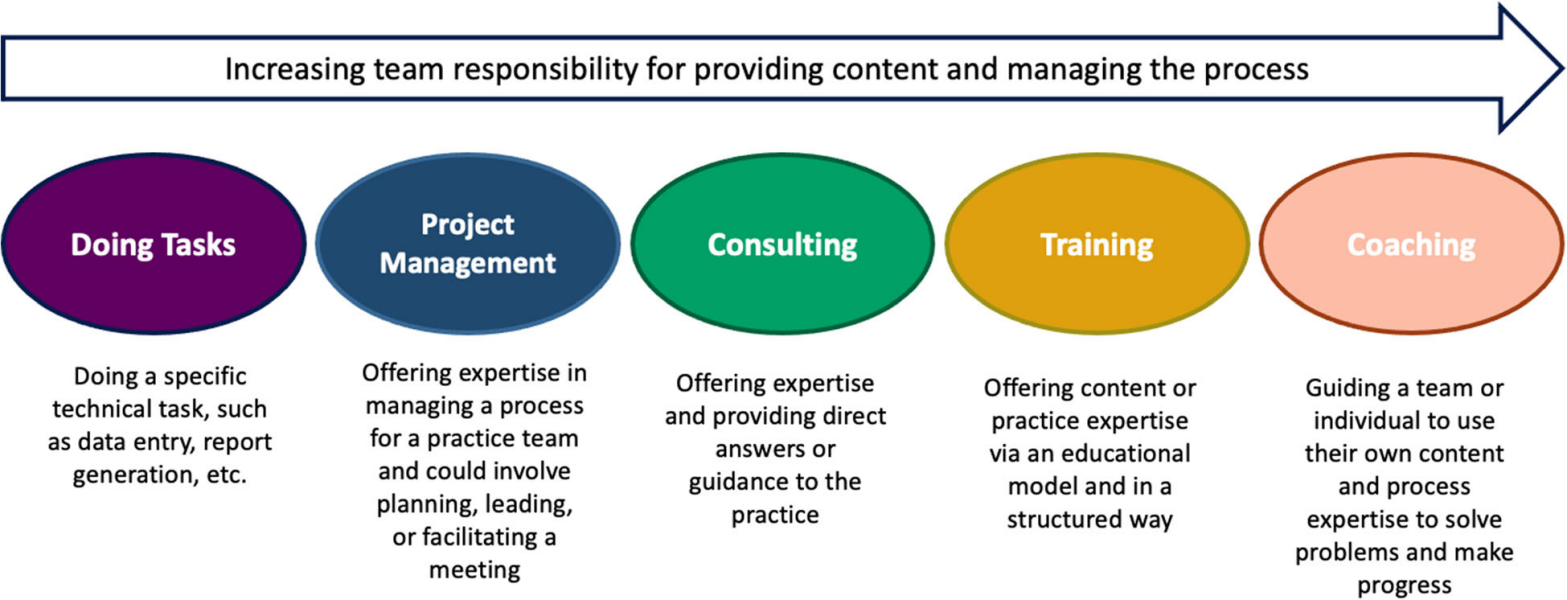
The Practice facilitation strategy spectrum framework as described by Baker et al. [30]

The Healthy Hearts in the Heartland (H3) study examined the role of practice facilitation on improvement of 4 cardiovascular clinical quality measures (CQM) in small primary care practices in Illinois, Indiana, and Wisconsin [27] as part of the Agency for Healthcare Research and Quality-funded *EvidenceNOW: Advancing Heart Health in Primary Care* program [28]. Overall, compared to baseline measures of the CQMs in the 226 participating practices, we saw a modest increase in all four measures after year-long practice facilitation support: 4% increases in aspirin use for ischemic vascular disease and controlling high blood pressure, and 5% increases in statin therapy for prevention and treatment of cardiovascular disease and tobacco use (smoking) screening and cessation. All increases were significant (*p* < 0.001), and importantly, improvements were sustained at 18 months, when facilitation had been absent for 6 months [29]. However, CQMs are a measure of practice- and patient-level outcomes while practice facilitators focus on supporting implementation of specific QI interventions to effect long-term practice change. Thus, our aim in this study was to understand the impact of practice facilitation on the implementation of QI interventions in H3 and the relationship between specific strategies used by practice facilitators and successful practice-level implementation of QI interventions. To do so, we applied a previously proposed framework [30] (Fig. 1) that classifies practice facilitation activities by the degree to which the practice relies on the facilitator versus develops its own process expertise. This framework posits that some activities lead to short-term progress but ultimately undermine sustainable change, such as tasks that are “done for” the practice. Other types of activities, such as coaching, help the practice in the long term by developing their internal capacity to enact and sustain change. While Baker et al. [30] reported anecdotal success in raising practice facilitators’ awareness of their activities and developing strategies that build long-term capacity, the framework has not been explored quantitatively. Given the interrelatedness of practice facilitation activities, we employed regression tree analysis, a supervised machine learning approach that can help to explain relationships between independent variables, to identify practice facilitation strategies that were associated with QI intervention implementation success and to assess the practice facilitation strategy profile that characterized practices that were able to implement the greatest number of QI interventions. In addition to describing the factors that contribute to successful QI intervention implementation, our ultimate goal was to explore whether the Baker et al. framework [30] could be a foundation for comparison of practice facilitation strategies across QI initiatives and the development of a common terminology for research into practice facilitation as a multicomponent implementation strategy.

## Methods

### Study population

The Healthy Hearts in the Heartland (H3) study was part of the EvidenceNOW initiative to improve cardiovascular care in small primary care practices in Illinois, Indiana, and Wisconsin, through practice facilitation [27]. As part of the EvidenceNOW program [28], AHRQ funded 7 projects around the country, all of which were required to use practice facilitation in service of implementing QI interventions. H3’s study design and practice characteristics have been previously described [27, 29]. Briefly, all participating practices were randomized to one of two study arms and the study was implemented in four sequential waves. Each practice was assigned a practice facilitator who received structured training on clinical topics and QI strategies related to the Million Hearts ABCS of heart health: Aspirin use for at-risk individuals, Blood pressure control, Cholesterol management, and Smoking cessation. Our study population comprised all 226 primary care practices recruited to participate in the study, including 26 practices that eventually withdrew. All practices were included in our analysis because all practices engaged in documented facilitation activities. As a retrospective study that included the entire practice population, we did not perform a power calculation, but in all power calculations for the primary H3 study design [27], we assumed a 2-sided type 1 error of 5% and anticipated adequate (80%) power to detect a meaningful difference between 5 and 10%.

### Tracking H3 practice facilitation activities and QI interventions

H3 used a tailored implementation practice facilitation strategy in which participating practices selected QI interventions to implement from a list of 35 that were designed for the study [27]. Examples of these interventions included reminders to order aspirin for patients with atherosclerotic cardiovascular disease and patient education on tobacco cessation. During a 12-month intervention period, practice facilitators worked with practice staff to implement their QI plan.

Within H3, we created a data collection tool to track the day-to-day activities of practice facilitators as they worked with practices and the study interventions the practices implemented. Practice facilitators recorded their activities with the practices in standardized fields using the Facilitation ACtivity and Intervention Tracking System (FACITS) tool [31], a modified customer relationship management system built using the QuickBase platform [32] to document recruitment and facilitation. FACITS was designed in collaboration with the practice facilitation team for H3 with an emphasis on collecting essential data elements for study evaluation and optimizing documentation efficiency. Each entry for interactions and/or tasks performed by practice facilitators was defined as an activity. At the time of entry, all activities were classified by practice facilitators as belonging to one or more of 27 standard practice facilitation activities designed for H3 that were pre-loaded into FACITS prior to study inception (Table S1). Examples of activities include engaging electronic health record (EHR) vendors, reviewing performance data with the practice, and coaching the practice to establish a care coordination process. For each activity performed, practice facilitators recorded the location, modality (e.g., in person, phone, virtual), start and end time of the engagement, practice staff who participated in the activity, and notes for follow-up.

At baseline and 12 months, practice facilitators reviewed progress with practice personnel on their selected QI interventions. Practice facilitators and practice staff met together to discuss progress or practices completed in an email form which was then reviewed by the facilitator. Practice facilitators were responsible for documenting interventions as “Complete” on FACITS if the practice had finished implementing the QI intervention at the time of review.

### Mapping H3 practice facilitation strategies to the spectrum of practice facilitation strategies

To investigate the relationship between the specific practice facilitation strategies practices engaged in and intervention implementation success in H3, 4 of the 13 (31%) practice facilitators systematically mapped H3 practice facilitation activities to a spectrum of practice facilitation strategies based on the framework described by Baker et al. [30] (Fig. 1). The 4 facilitators had leadership roles, represented all 3 states (Illinois, Indiana, and Wisconsin), and 4 (Northwestern University, Purdue University, the American Medical Association and Metastar) of the 7 organizations that provided practice facilitation. Telligen, AllianceChicago, and Northern Illinois University practice facilitators did not participate in the mapping activity.

After being instructed on the practice facilitation spectrum and reviewing examples of the overall strategies as described by Baker et al. [30], the 4 facilitators each independently mapped the 27 H3 standard facilitation strategies to Baker et al.’s 5 practice facilitation strategy categories: Doing Tasks, Project Management, Consulting, Teaching, and Coaching (Fig. 1). To ensure clear nomenclature, we changed the name of the category originally described by Baker et al. as “Facilitating” to “Project Management”. One practice facilitator (JB) was charged with reviewing the all the facilitators’ individual maps and identifying conflicts. Conflicts were resolved through majority vote. If no majority was present, practice facilitators conferenced until reaching unanimous decision.

### Statistical analysis

Correlation analyses were performed to investigate relationships between inputs (the total number of practice facilitation activities and the number of times each practice facilitation strategy performed) and the primary outcome (the number of QI interventions marked as “Completed” at the end of the tracking period). During the context of any visit, practice facilitators may engage in practice facilitation activities that are part of one or more strategies. For instance, a practice facilitator could help coordinate the next meet of the QI team (Project Management), provide education on the implementation of a specific intervention (Training), and participate in a practice-level review of quality measures a team member extracted from their medical record system to help assess measure improvement (Coaching). Thus, to understand the relationship between practice facilitation strategy variables, we chose a multi-variable analysis strategy that could represent the interrelated nature of the activities performed by the practice facilitators. Regression tree analysis, a form of supervised sub-grouping commonly used in machine learning, recursively partitioned the practices into mutually exclusive groupings based on the frequency of strategies. This resulted in a set of practice groups with minimal intra-group variation and maximal inter-group variation of completed interventions [33–35]. Regression tree algorithms select features and determine cutoffs for splitting groups into sub-groups that would result in the best partition in terms of the variance, resulting in two sub-groups that have the greatest degree of difference with regard to the target outcome, and add this split to the tree. Compared with traditional linear models, regression trees result in output rules that explain hierarchical and linear relationships between variables, are easy to interpret (and thus, use in the context of developing future practice facilitation interventions), and provide intuitive visualization of predictors that have the greatest impact on subgroup identification.

We used Kruskal-Wallis tests to identify whether the sub-groups identified in the regression tree analysis correlated with the primary outcome and Kruskal-Wallis tests and chi-squared tests of association to explore whether practice characteristics, geographic location, number of practice facilitator visits, intervention duration, and study completion differed across the sub-groups.

## Results

**Fig. 2:**
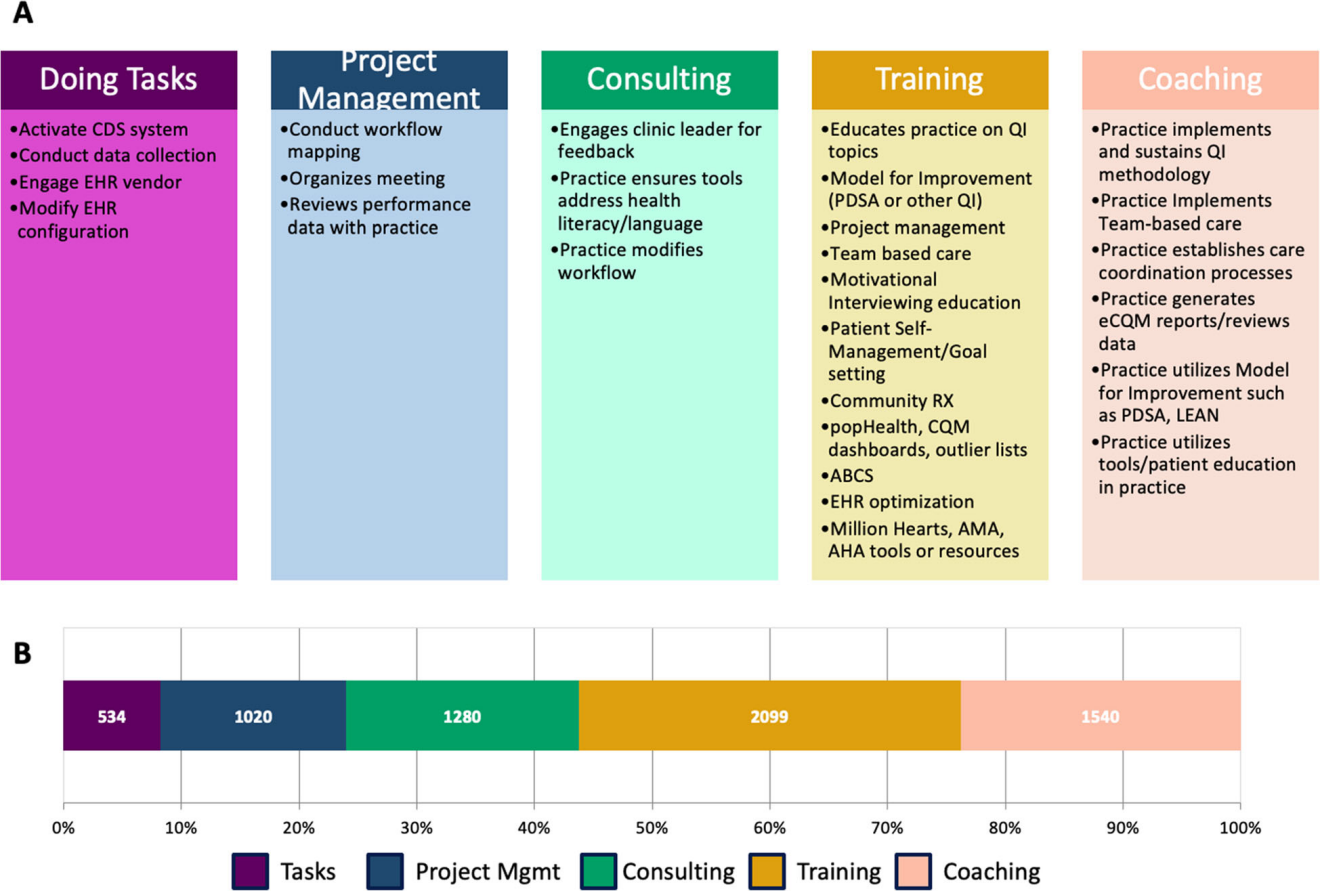
**a** H3 standard facilitation actvities mapped to the spectrum of practice facilitation strategies. **b** Number and frequency of strategies across all practices experienced by all 226 practices in the H3 study

The experienced practice facilitators successfully mapped all 27 H3 standard practice facilitation activities to the 5 overarching practice facilitation strategies (Fig. 2a). Independently, all 4 practice facilitators identically mapped 24 (88.9%) activity categories. One of the 4 practice facilitators had a discrepancy on the remaining 3 (11.1%) activity categories: practice ensures tools address health literacy/language, practice modifies workflow, and practice utilizes tools/patient education in practice. Since 3 practice facilitators had consensus in their independent maps, these 3 activity categories were assigned to the strategies agreed upon by the majority. Due to high concordance among practice facilitators, no activity categories required conferencing to determine assignment.

Of the 4485 documented activity instances that the 226 practices in the study participated in, Training strategies were the most prevalent (32.4%), followed by Coaching (23.8%), Consulting (19.8%), Project Management (15.8%), and Doing Tasks (8.2%) (Fig. 2b).

**Fig. 3:**
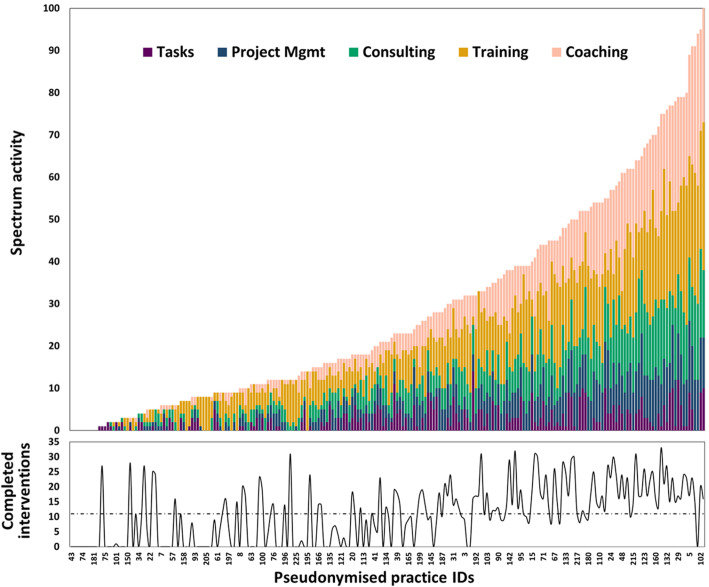
Frequency of practice facilitation activities (grouped by practice facilitation strategy) experienced by practices in H3 and completed interventions by practices in H3

Figure 3 shows the distribution of activities in each practice, sorted by number of activities and practices’ corresponding completed QI interventions over the course of the year-long intervention period. The total number of activities varied widely among practices, with practices participating in a median of 21 practice facilitation activities (interquartile range (IQR) 9 to 44). The number and proportion of strategies also varied widely among practices. While all practices engaged in at least 1 activity with their facilitator, and most (91.1%) practices participated in activities of each of the five overarching strategy types, only 66.4% of practices completed any QI intervention, again with substantial variation. Practices implemented a median of 10 interventions (out of 35 possible), ranging from 0 to 35 completed interventions. Using linear regression analysis, we found no correlation between the total number of activities the practices participated in with their practice facilitators and the number of QI interventions the practices ultimately completed (*β* = 0.04, *r*^2^ = 0.33, *p* = 0.47). In correlation analyses of proportions of each strategy with completed interventions, the proportion of activities that were considered Project Management strategies (as defined in Figs. 1 and 2, where facilitators would share their organizational expertise to coordinate activities in the practice such as initiating team meetings) were positively associated with completed interventions (*β* = 1.05, *r*^2^ = 0.34, *p* < 0.001). However, there was no significant correlation between the proportion of activities in any other strategy category and the number of successfully implemented QI interventions.

**Fig. 4:**
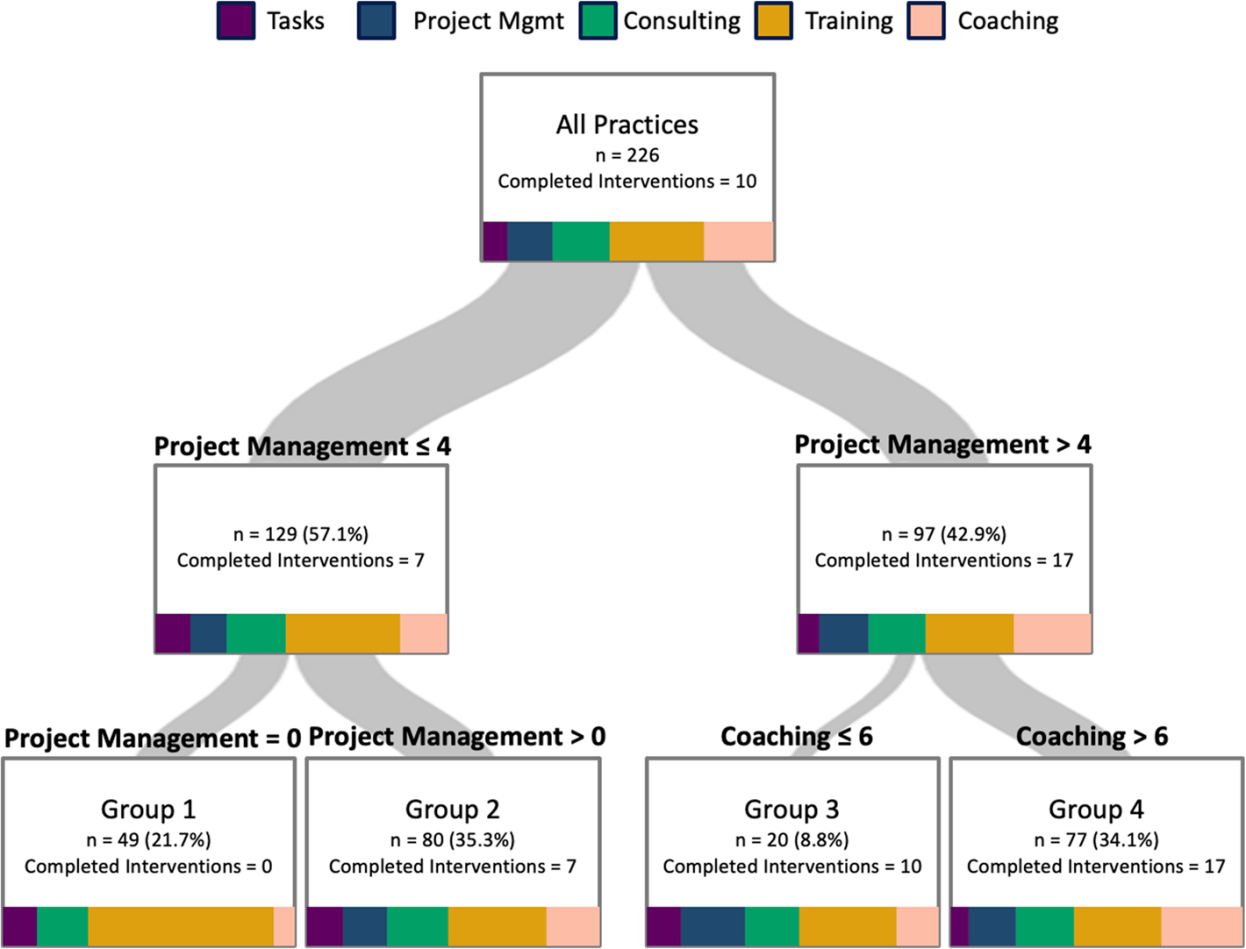
Regression tree analysis for the spectrum of practice facilitation strategies experienced by the 226 practices in the H3 study. Spectrum pictures in subgroup boxes represent the distribution of the activities received by practices in that subgroup, grouped by strategy type

We next employed regression tree analysis, a supervised machine learning strategy, to better understand the relationship of individual strategies, as well as the interactions between the strategies, with successful implementation of cardiovascular care QI interventions in H3. This analysis assessed associations of the 5 overall strategy types with the number of implemented interventions at 12 months, the end point of the study. One benefit of regression tree analysis is that each branching of the tree provides a defined, understandable rule by which the branch was made. The regression tree ultimately split the 226 practices into 4 groups (Fig. 4), with the first rule subdividing the practices based on the number of Project Management strategy activities the practices participated in, and the second rule further subdividing the groups by Project Management strategy activities and Coaching activities participated in. Group 1, the lowest performing group based on interventions implemented, included 49 (22%) practices that participated in < 1 Project Management activity and implemented a median of 0 interventions. The 80 (35%) practices in group 2 participated in 1 to 4 Project Management activities and implemented a median of 7 interventions. Group 3 had 20 (9%) practices that participated in > 4 Project Management activities and ≤ 6 Coaching activities and implemented a median of 10 interventions. Finally, the highest performing group (group 4) included 77 (34%) practices that participated in > 4 Project Management activities and > 6 Coaching activities and implemented a median of 17 of 35 interventions. We depicted the distribution of strategies that each group participated in to provide additional information about the relationship between practice facilitation strategies and group. This is represented on each segment in the tree using the same visualization as Fig. 2b. For instance, practices in group 1 were the only group that participated in no Project Management activities, the fewest Coaching activities, and the largest proportion of Training activities, while practices in group 4 participated in practice facilitation activities of every strategy type and the largest proportion of Coaching activities of any group in the whole practice population.

**Fig. 5:**
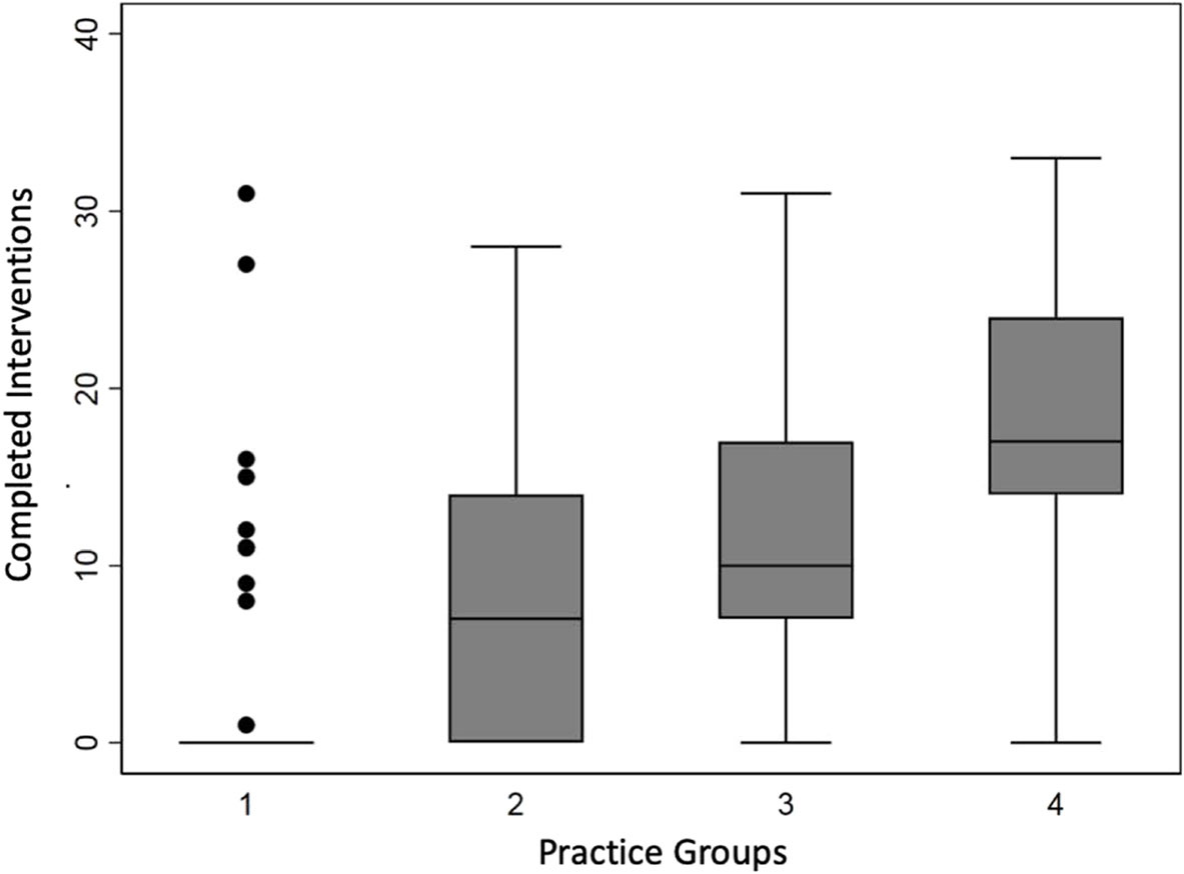
Box plot of the distribution of interventions completed by practices participating in the H3 study as assessed by regression tree segmentation

The boxplot in Fig. 5 describes the distribution of successfully implemented interventions by practices in each of the 4 final groups. The 4 groups had markedly different degrees of variation in implemented interventions at 12 months, from a median of 0 implemented interventions in group 1, increasing stepwise to a median of 17 in group 4.

To further understand how practice and study characteristics related to the groups identified using our supervised machine learning strategy, we assessed the distribution of practice characteristics (geographic location, number of physicians, study wave, participation in a practice network or designation as a Federally Qualified Health Center (FQHC)) and study characteristics (number of facilitator visits, intervention duration, and study completion) across all the groups as compared to the whole population of 226 practices that participated in the study. The results of these analyses are shown in Table 1. Groups did not differ significantly by study wave, practice size, association with a larger healthcare network, nor FQHC designation. Within the practice characteristics assessed, only geographic location was statistically significantly different compared to the full cohort, where practices in Wisconsin were proportionally overrepresented in group 4, the highest performing group with regard to the implemented interventions. Within the study characteristics investigated, the linked attributes of the number of facilitator visits and intervention durations increased across the groups, with group 1 and group 4 practices receiving the smallest (3 visits IQR [1–4], 120 min IQR [90–188]) and largest (8 visits IQR [7–10], 455 min IQR [330–630)]) number of visits and number of minutes, respectively. The differences observed were statistically significantly different from the full practice population. Finally, a significant difference was observed between the proportion of practices dropping out of the study across the 4 groups compared to the population as a whole. In particular, 9 of the 13 that terminated early were part of group 1, the group with the overall smallest number of successfully implemented interventions.

**Table 1:** Practice characteristics, number of visits, intervention duration, and study completion by regression tree sub-group

	Total (*N*=226)	Group 1 (*N*=49)	Group 2 (*N*=80)	Group 3 (*N*=20)	Group 4 (*N*=77)	*P* value
**State**, *N* (%)						0.004
IL	153 (67.7)	35 (71.4)	61 (76.3)	16 (80.0)	41 (53.3)	
IN	51 (22.6)	12 (24.5)	15 (18.7)	2 (10.0)	22 (28.6)	
WI	22 (9.7)	2 (4.1)	4 (5.0)	2 (10.0)	14 (18.2)	
**Wave**						0.81
1	42 (18.6)	9 (18.4)	14 (17.5)	4 (20.0)	15 (19.5)	
2	40 (17.7)	9 (18.4)	16 (20.0)	4 (20.0)	11 (14.3)	
3	67 (29.7)	10 (20.4)	21 (26.3)	6 (30.0)	30 (39.0)	
4	77 (34.1)	21 (42.9)	29 (36.3)	6 (30.0)	21 (27.3)	
**Number of clinicians**, *N* (%)						0.95
Solo	89 (39.4)	19 (38.8)	34 (42.5)	8 (40.0)	28 (36.4)	
2–5	116 (51.3)	24 (49.0)	38 (47.5)	9 (45.0)	45 (58.4)	
6–10	19 (8.4)	4 (8.2)	8 (10.0)	3 (15.0)	4 (5.2)	
11–15	2 (0.9)	2 (4.1)	0	0	0	
**Network,** ***N*** **(%)**						0.76
Yes	76 (33.6)	21 (42.9)	28 (35.0)	6 (30.0)	21 (27.3)	
No	150 (66.4)	28 (57.1)	52 (65.0)	14 (70.0)	56 (72.7)	
**FQHC,** ***N*** **(%)**						0.96
Yes	64 (28.3)	16 (32.7)	24 (30.0)	5 (25.0)	19 (24.7)	
No	162 (71.7)	33 (67.4)	56 (70.0)	15 (75.0)	58 (75.3)	
**Visits**, median (IQR)	5 (3–8)	3 (1–4)	4 (3–5)	8 (5–9.5)	8 (7–10)	< 0.001
**Duration**, median (IQR)	305 (165–495)	120 (90–188)	250 (150–338)	373 (280–773)	455 (330–630)	< 0.001
**Completed study**						< 0.001
Yes	213 (94.3)	40 (81.6)	77 (96.3)	20 (100.0)	76 (98.7)	
No	13 (5.7)	9 (18.4)	3 (3.75)	0	1 (1.3)	

## Discussion

To assess the impact of different discrete aspects of the multicomponent practice facilitation strategy on the implementation of QI interventions in the H3 study, we mapped 27 unique activities performed by the H3 practice facilitators across a spectrum [30] that ranged from Doing Tasks for the practices to Coaching the practices on their identified goals. While 11 of the 27 standard practice facilitation activities in H3 were identified as training strategies, the H3 study had practice facilitation activities in every strategy category in the spectrum, and almost 80% of practices in the study experienced practice facilitation support in all 5 overarching strategy types. Additionally, almost a quarter of the activities performed with H3 practices were focused on Coaching, the strategy type believed to be optimal for effecting sustained practice change [30].

Surprisingly, we did not observe any correlation between the total number of practice facilitation activities practices participated in and the number of QI interventions practices implemented during the study. However, using regression tree analysis, we demonstrated that participating in more Project Management and Coaching strategy activities was associated with an increased number of implemented QI interventions at the end of 12 months, with practices that participated in > 4 Project Management activities and > 6 Coaching activities implementing a median of 17 of 35 interventions. This suggests that strategies that emphasize the development of internal practice capacity are associated with success in implementing QI interventions in our study and that the framework proposed by Baker et al. [30] was an effective organizing structure for assessing facilitation strategies within the context of our study.

Practice context, in addition to practice facilitation activity, is likely to have an impact on successful implementation of QI interventions. Thus, we also assessed the relationship of several key practice context variables to the practice groups identified through regression tree analysis, including geographic region of the practice, study wave, practice type, and whether the practice was part of a healthcare network. Of these variables, only geographic location was associated with sub-grouping. It remains to be determined whether the association with location was due to state-level programs, such as support for patient-centered medical home, or the practice facilitation team working in the location (as H3 practice facilitators worked primarily within a single state). Interestingly, there was no association of study wave, practice size, practice type, or participation in a health network with the groups from the regression tree analysis. This suggests that the experience the practice facilitators developed over the course of H3 did not lead to improved practice outcomes in later waves of the study. Also of note, regardless of size, type, network participation, or FQHC designation, practices that participated in more activities based on Coaching strategies were more likely to implement more QI interventions during the study, suggesting that a variety of practice team compositions and resourcing levels may be the foundation for successful implementation of QI interventions when practices participate in more coaching focused activities delivered by practice facilitators.

While a previously published assessment of our study [29] found no association between the overall duration of the practice facilitation, as measured by number of minutes or number of visits, and overall study clinical quality measure outcomes (e.g., improvement in the Million Hearts ABCS clinical quality measures), we saw a distinct association between number and duration of visits with practice groups that participated in more coaching and completed more QI interventions, consistent with the systematic review of facilitation activities performed by Baskerville et al. [8]. Likewise, practices that participated in more activities under the Project Management and Coaching strategy categories were more likely to complete the study. Understanding whether the additional participation in coaching activities was driven by the practice facilitator, the internal context of the practice, or resulted from the dynamic interactions of both facilitator and practice attributes remains to be elucidated through more complicated modeling activities.

### Implications for implementation science

This study used a novel statistical method that can shed light on a challenging area of implementation science related to the reporting, specification, and impact of specific strategies. Reporting guidelines put forth by Proctor et al. [22] were a significant step in attempting to move the field toward more uniform and complete descriptions of the strategies being tested and observed. Proper specification permits research synthesis and drawing generalizable conclusions regarding the effectiveness of particular strategies for certain innovations under specific contextual circumstances. However, a number of recent systematic reviews and meta-analyses that have attempted to do just this have been hampered due to poor specification of the strategy being investigated [36, 37]. This concern is further underscored by a second challenge for the field, which is understanding the active ingredients of multicomponent strategies (i.e., those that combine multiple discrete strategies) that drive change. Also referred to as multifaceted, blended, or bundled strategies and implementation interventions, these often protocolized “packages” of strategies are both difficult to report accurately given the inevitable heterogeneity of specific use across sites and across time (within and between sites) and to disentangle when it comes to identifying the discrete strategies responsible for observed changes in implementation outcomes. This issue is not limited to practice facilitation, but it is a prime example of a multicomponent strategy that is commonly used and has been shown to be highly variable within and across studies [8, 38].

The regression tree analysis approach used in this study demonstrates an innovative means of understanding the relative contribution of discrete strategies within larger multicomponent implementation strategies. An approach using machine learning can reveal relationships and hierarchical patterns that might be obscured by more traditional regression-based models that require a human driven model-building approach. In essence, traditional methods attempt to “fit” the data to a hypothesized model, which is inherently limited to the way researchers conceptualize the relationship between variables, whereas machine learning approaches, with minimal or no guidance from the analyst, can determine more complex patterns and structures in the data [39]. Given that implementation research occurs in complex multilevel systems, these types of analytic models are likely to prove more and more useful to the field.

Practically speaking, resource limitations are inevitably of concern in implementation research, particularly in under-resourced/resource-limited healthcare delivery systems. While potentially effective, multicomponent implementation strategies are costly and time consuming for clinical practices, and it is critical that they be streamlined to be time and cost-effective and minimize the magnitude of capital investment and personnel time for most implementing entities. This study revealed that some strategies within practice facilitation may be less effective or have a weaker relationship with driving the implementation of interventions and should be further evaluated in other similar studies to determine whether pruning of activities could optimize practice facilitation in studies similar to H3.

### Limitations and future directions

Our study has several limitations. Our dataset, while large, represents one study focused on improving cardiovascular care for adult patients. More studies where practice facilitation activities are mapped to the Facilitation Strategy Spectrum, particularly studies with different types of QI goals, are needed to evaluate the performance and generalizability of the framework to other studies of practice facilitation-driven implementation. Likewise, our practice facilitation activities were mapped to practice facilitation strategy categories by a group of practice facilitators with deep knowledge of H3. A broader group of practice facilitators may have mapped the activities to different strategies or the activities in the context of a different study could have been evaluated to support different strategies. This speaks to the greater need to develop a standardized framework and terminology system to support the analysis of QI implementation strategies in general and within practice facilitation specifically. Finally, we assessed only a small subset of contextual variables in relationship to our practice grouping analysis. It is probable that clinical leadership and engagement, participation in previous QI activities, and significant change in the practice (such as rip and replace of an EHR system or personnel changes) could also have a relationship to the responsiveness to practice facilitation activities as well as the individual capabilities of the practice facilitators themselves.

## Conclusions

Taken together, our data suggest that practices that participate in more practice facilitation activities characterized under the broader umbrella categories of Project Management and Coaching strategies implement more QI interventions for cardiovascular health, and practice receptivity to these strategies is not associated with practice size, type, or participation in a larger healthcare network. We also demonstrated that that the practice facilitation strategy spectrum introduced by Baker et al. [30] may be an effective foundation for building a framework whereby study-specific practice facilitation activities can be mapped to broader implementation strategy categories to assess practice facilitation success using supervised machine learning approaches. This suggests that development of a standardized informatics-driven data framework and terminology system to describe practice facilitation activities could be an effective infrastructure to support cross-study comparisons of practice facilitation strategies and the relationship between these strategy types on intervention outcomes, which in turn could grow our understanding of how practice facilitation can be most effective in supporting improved quality of care across a variety of practice settings and intervention types. This study contributes to the ongoing push in the field to improve specification of implementation strategies and to better understand precisely what leads to the effectiveness of multicomponent and discrete implementation strategies at improving care.

## Supplementary Information

**Additional file 1: Table S1.** H3 Intervention Activities and Detailed Definitions of the Activities.

## Data Availability

The datasets analyzed during this study are not publicly available given privacy protections for individual level data collected for the study and the potential for re-identification of practice participants. Datasets are available from the corresponding author on reasonable request and completion of appropriate data sharing agreements.

## Abbreviations

EHR: Electronic health record
FACITS: Facilitation ACtivity and Intervention Tracking System
FQHC: Federally Qualified Health Center
H3: Healthy Hearts in the Heartland Study
IQR: Interquartile range
PF: Practice facilitator
QI: Quality improvement

## References

[1] Geonnotti K, Taylor EF, Peikes D, Schottenfeld L, Burak H, McNellis R (2015). Engaging primary care practices in quality improvement: strategies for practice facilitators.

[2] Nutting PA, Crabtree BF, McDaniel RR (2012). Small primary care practices face four hurdles--including a physician-centric mind-set--in becoming medical homes. Health Aff (Millwood).

[3] Cohen MF (2016). Impact of the HITECH financial incentives on EHR adoption in small, physician-owned practices. Int J Med Inform.

[4] Kenefick H, Lee J, Fleishman V (2008). Improving physician adherence to clinical practice guidelines: barriers and strategies for change.

[5] McHugh M, Brown T, Walunas TL, Liss DT, Persell SD (2019). Contrasting perspectives of practice leaders and practice facilitators may be common in quality improvement initiatives. J Healthc Qual.

[6] Taylor EF, Machta RM, Meyers DS, Genevro J, Peikes DN (2013). Enhancing the primary care team to provide redesigned care: the roles of practice facilitators and care managers. Ann Fam Med.

[7] Liddy CE, Blazhko V, Dingwall M, Singh J, Hogg WE (2014). Primary care quality improvement from a practice facilitator’s perspective. BMC Fam Pract.

[8] Baskerville NB, Liddy C, Hogg W (2012). Systematic review and meta-analysis of practice facilitation within primary care settings. Ann Fam Med.

[9] Wang A, Pollack T, Kadziel LA, Ross SM, McHugh M, Jordan N, Kho AN (2018). Impact of practice facilitation in primary care on chronic disease care processes and outcomes: a systematic review. J Gen Intern Med.

[10] Dickinson WP, Dickinson LM, Nutting PA, Emsermann CB, Tutt B, Crabtree BF, Fisher L, Harbrecht M, Gottsman A, West DR (2014). Practice facilitation to improve diabetes care in primary care: a report from the EPIC randomized clinical trial. Ann Fam Med.

[11] Meropol SB, Schiltz NK, Sattar A, Stange KC, Nevar AH, Davey C, Ferretti GA, Howell DE, Strosaker R, Vavrek P, Bader S, Ruhe MC, Cuttler L (2014). Practice-tailored facilitation to improve pediatric preventive care delivery: a randomized trial. Pediatrics..

[12] Frost MC, Ioannou GN, Tsui JI, Edelman EJ, Weiner BJ, Fletcher OV, Williams EC (2020). Practice facilitation to implement alcohol-related care in Veterans Health Administration liver clinics: a study protocol. Implement Sci Commun.

[13] Kirchner JE, Ritchie MJ, Pitcock JA, Parker LE, Curran GM, Fortney JC (2014). Outcomes of a partnered facilitation strategy to implement primary care-mental health. J Gen Intern Med.

[14] Siantz E, Redline B, Henwood B (2020). Practice facilitation in integrated behavioral health and primary care settings: a scoping review. J Behav Health Serv Res.

[15] Weiner BJ, Rohweder CL, Scott JE, Teal R, Slade A, Deal AM (2017). Using practice facilitation to increase rates of colorectal cancer screening in community health centers, North Carolina, 2012-2013: feasibility, facilitators, and barriers. Prevent Chron Dis.

[16] Quinn JM, Gephart SM, Davis MP (2019). External facilitation as an evidence-based practice implementation strategy during an antibiotic stewardship collaborative in neonatal intensive care units. Worldviews Evid-Based Nurs.

[17] Buscaj E, Hall T, Montgomery L, Fernald DH, King J, Deaner N (2016). Practice facilitation for PCMH implementation in residency practices. Fam Med.

[18] Harder VS, Long WE, Varni SE, Samuelson J, Shaw JS (2017). Pediatric-informed facilitation of patient-centered medical home transformation. Clin Pediatr (Phila).

[19] Nutting PA, Crabtree BF, Stewart EE, Miller WL, Palmer RF, Stange KC (2010). Effect of facilitation on practice outcomes in the National Demonstration Project model of the patient-centered medical home. Ann Fam Med.

[20] Crabtree BF, Nutting PA, Miller WL, McDaniel RR, Stange KC, Jaen CR (2011). Primary care practice transformation is hard work: insights from a 15-year developmental program of research. Med Care.

[21] Nagykaldi Z, Mold JW, Robinson A, Niebauer L, Ford A (2006). Practice facilitators and practice-based research networks. J Am Board Fam Med.

[22] Proctor EK, Powell BJ, McMillen JC (2013). Implementation strategies: recommendations for specifying and reporting. Implement Sci.

[23] Powell BJ, Fernandez ME, Williams NJ, Aarons GA, Beidas RS, Lewis CC, McHugh SM, Weiner BJ (2019). Enhancing the impact of implementation strategies in healthcare: a research agenda. Front Public Health.

[24] Powell BJ, Waltz TJ, Chinman MJ, Damschroder LJ, Smith JL, Matthieu MM, Proctor EK, Kirchner JAE (2015). A refined compilation of implementation strategies: results from the Expert Recommendations for Implementing Change (ERIC) project. Implement Sci.

[25] Bunger AC, Powell BJ, Robertson HA, MacDowell H, Birken SA, Shea C (2017). Tracking implementation strategies: a description of a practical approach and early findings. Health Res Policy Syst.

[26] Huynh AK, Hamilton AB, Farmer MM, Bean-Mayberry B, Stirman SW, Moin T, Finley EP (2018). A pragmatic approach to guide implementation evaluation research: strategy mapping for complex interventions. Front Public Health.

[27] Ciolino JD, Jackson KL, Liss DT, Brown T, Walunas TL, Murakami L, Chung I, Persell SD, Kho AN (2018). Design of healthy hearts in the heartland (H3): a practice-randomized, comparative effectiveness study. Contemp Clin Trials.

[28] EvidenceNOW: advancing heart health in primary care: Agency for Healthcare Research and Quality; [Available from: https://www.ahrq.gov/evidencenow/index.html.

[29] Persell SD, Liss DT, Walunas TL, Ciolino JD, Ahmad FS, Brown T, French DD, Hountz R, Iversen K, Lindau ST, Lipiszko D, Makelarski JA, Mazurek K, Murakami L, Peprah Y, Potempa J, Rasmussen LV, Wang A, Wang J, Yeh C, Kho AN (2020). Effects of 2 forms of practice facilitation on cardiovascular prevention in primary care: a practice-randomized, comparative effectiveness trial. Med Care.

[30] Baker N, Lefebvre A, Sevin C (2017). A framework to guide practice facilitatators in building capacity. J Fam Med Commun Health.

[31] Ye J, Zhang R, Bannon JE, Wang AA, Walunas TL, Kho AN, Soulakis ND (2020). Identifying practice facilitation delays and barriers in primary care quality improvement: a report from Evidence NOW. J Am Board Fam Med.

[32] QuickBase [Available from: https://www.quickbase.com/.

[33] Prasad AM, Iverson LR, Liaw A (2006). Newer classification and regresssion tree techniques: bagging and random forests for ecological prediction. Ecosystems..

[34] Schilling C, Mortimer D, Dalziel K, Heeley E, Chalmers J, Clarke P (2016). Using Classification and Regression Trees (CART) to identify prescribing thresholds for cardiovascular disease. Pharmacoeconomics..

[35] Crowe S, Brown K, Tregay J, Wray J, Knowles R, Ridout DA, Bull C, Utley M (2017). Combining qualitative and quantitative operational research methods to inform quality improvement in pathways that span multiple settings. BMJ Qual Saf.

[36] Ivers NM, Grimshaw JM, Jamtvedt G, Flottorp S, O’Brien MA, French SD, Young J, Odgaard-Jensen J (2014). Growing literature, stagnant science? Systematic review, meta-regression and cumulative analysis of audit and feedback interventions in health care. J Gen Intern Med.

[37] Garcia-Elorrio E, Rowe SY, Teijeiro ME, Ciapponi A, Rowe AK (2019). The effectiveness of the quality improvement collaborative strategy in low- and middle-income countries: a systematic review and meta-analysis. PLoS One.

[38] Nguyen AM, Cuthel A, Padgett DK, Niles P, Rogers E, Pham-Singer H, Ferran D, Kaplan SA, Berry C, Shelley D (2020). How practice facilitation strategies differ by practice context. J Gen Intern Med.

[39] Brown CH, Mohr DC, Gallo CG, Mader C, Palinkas L, Wingood G, Prado G, Kellam SG, Pantin H, Poduska J, Gibbons R, McManus J, Ogihara M, Valente T, Wulczyn F, Czaja S, Sutcliffe G, Villamar J, Jacobs C (2013). A computational future for preventing HIV in minority communities: how advanced technology can improve implementation of effective programs. JAIDS J Acquired Immune Def Syndr.

