# Supplementary material for: Does coaching matter? Examining the impact of specific practice facilitation strategies on implementation of quality improvement interventions in the Healthy Hearts in the Heartland study
Source: Implement Sci. 2021 Mar 31;16:33. doi: 10.1186/s13012-021-01100-8 (PMC8011080; doi:10.1186/s13012-021-01100-8)
Supplement: Supplementary file 1 — Additional file 1: Table S1. H3 Intervention Activities and Detailed Definitions of the Activities. [file 13012_2021_1100_MOESM1_ESM.docx]

Table S1: H3 Intervention Activities and Detailed Definitions of the Activities

| H3 interventions | Definition |
| --- | --- |
| 1. Facilitator Activates Clinical decision support systems | Implementation of clinical decision support EMR optimization |
| 2. Facilitator Conducts data collection | Aggregate report on ABCS or manual chart extraction by facilitator |
| 3. Facilitator Engaged clinic leader for feedback | Bi-directional communication between clinic leader and facilitator |
| 4. Facilitator Educates practice on QI topics | Topics include goal setting, data tracking over time, transparency |
| 5. Facilitator Reviews data with practice for performance monitoring | ABCS data outcomes shared with practice |
| 6. Facilitator Engages EHR vendor re: identified issues | Facilitator acts as direct liaison between vendor and practice |
| 7. Facilitator Modifies EHR configuration | EHR configured to generate ABCS clinical quality measures to collect aggregate outcome variables |
| 8. Facilitator conducts Workflow mapping | Document roles and responsibilities of all practice staff |
| 9. Facilitator organizes meeting | Arrangement of date, time, place and attendees |
| 10. Train Model for Improvement (PDSA or other QI) | Instruction on rapid cycle model, pilot testing, data tracking |
| 11. Train Project management | Educate on study goals, timeline and success criteria |
| 12. Train Team based care (huddles pre/post visit planning) | Model in which two or more individuals work collaboratively to provide care |
| 13. Train Motivational Interviewing education | Skills-based model of interactive communication strategies to elicit change |
| 14. Train Patient Self- Management/Goal setting | Includes shared decision-making tools, Patient action plans |
| 15. Train Community RX | Customized tool containing community resources based on geographic location |
| 16. Train popHealth, CQM dashboards, outlier lists | Utilize data to assist with outreach of patients not in numerator |
| 17. Train ABCS | Evidence-based training on ABCS measures |
| 18. Train EHR optimization | Educate staff on CDS, Reminders, Templates |
| 19. Train Million Hearts, AMA, AHA tools or resources | Evidence-based resources developed by American Medical Association, American Heart Association |
| 20. Practice implements and sustains QI methodology | QI methods include data tracking, review and transparency |
| 21. Practice Implements Team-based care | Model in which two or more individuals work collaboratively to provide care |
| 22. Practice establishes care coordination processes | Organization of activities to facilitate care delivery |
| 23. Practice ensures tools address health literacy/language | Resources/tools utilized in multiple languages, literacy levels |
| 24. Practice generates eCQM reports/reviews data | Independent report generation of ABCS outcome measures by practice |
| 25. Practice modifies workflow | Roles and responsibilities of all practice staff modified to facilitate improved patient care delivery |
| 26. Practice utilizes Model for Improvement such as PDSA, LEAN | Instruction on rapid cycle model, pilot testing, data tracking |
| 27. Practice utilizes tools/patient education in practice | Education/Tools on ABCS resources distributed electronic or manual |
